# Horizontal Gene Transfer in *Listeria monocytogenes*: Evolution of Antimicrobial Resistance and Virulence in a One Health Context

**DOI:** 10.3390/biology15120961

**Published:** 2026-06-19

**Authors:** Georgeta Stefan, Maria Rodica Gurau, Nicoleta Ciocîrlie, Laurențiu Tudor, Stelian Bărăităreanu, Diana-Lidia Tache-Codreanu, Corina Sporea, Alexandru Gligor, Ionica Iancu, Viorel Herman

**Affiliations:** 1 Faculty of Veterinary Medicine, University of Agronomic Sciences and Veterinary Medicine of Bucharest, 011464 Bucharest, Romania; georgeta.stefan@fmvb.usamv.ro (G.S.); nicoleta.ciocirlie@fmvb.usamv.ro (N.C.); laurentiu.tudor@fmvb.usamv.ro (L.T.); stelian.baraitareanu@fmvb.usamv.ro (S.B.); 2 Faculty of Medicine, University “Titu Maiorescu”, 040441 Bucharest, Romania; dianatache@yahoo.com; 3 Faculty of Midwifery and Nursing, University of Medicine and Pharmacy ”Carol Davila”, 020021 Bucharest, Romania; corina.sporea@gmail.com; 4 Faculty of Veterinary Medicine, University of Life Sciences “King Mihai I” from Timisoara, 300645 Timisoara, Romania; ionica.iancu@usvt.ro (I.I.); viorel.herman@fmvt.ro (V.H.); 5 Academy of Romanian Scientists (AOSR), Street Ilfov, Number 3, Sector 5, 50044 Bucharest, Romania

**Keywords:** *Listeria monocytogenes*, horizontal gene transfer, antimicrobial resistance, virulence, mobilome, population genomics, one health

## Abstract

*Listeria monocytogenes* is a ubiquitous Gram-positive bacterium responsible for listeriosis, a foodborne zoonotic disease affecting humans and animals. Although infection in immunocompetent individuals is often asymptomatic or limited to mild self-limiting gastroenteritis, *Listeria monocytogenes* may cause severe invasive disease in vulnerable groups, including pregnant women, neonates, elderly individuals, and immunocompromised patients. Although the incidence of listeriosis is relatively low compared with many other foodborne pathogens, the high hospitalization and mortality rates associated with clinical cases make this bacterium a major concern for food safety and public health. The evolutionary success of *L. monocytogenes* reflects the interaction between a conserved core genome and a dynamic accessory genome shaped by horizontal gene transfer (HGT), ecological selection, and expansion of specific clones. Transient intestinal carriage in humans and animals, potentially influenced by gut microbiome composition, creates ecological interfaces where plasmids, transposons, prophages, and integrative conjugative elements contribute to the exchange of antimicrobial resistance determinants, virulence factors, and stress tolerance systems. Virulence diversification is further influenced by the differential distribution of pathogenicity islands such as LIPI-1, LIPI-3, and LIPI-4 across specific clonal lineages. These evolutionary processes occur across interconnected farm, food-production, environmental, and clinical ecosystems consistent with the One Health framework. Advances in whole-genome sequencing have clarified lineage-specific gene flow, expansion of specific clones, and the dynamics of the resistome and mobilome in *L. monocytogenes* populations. This narrative review aims to synthesize current knowledge on the mobile genetic elements and ecological interfaces that shape horizontal gene transfer in *L. monocytogenes*. Its novelty lies in integrating antimicrobial resistance, virulence-associated genomic islands, stress adaptation, and gut microbiome-mediated selection within a One Health and metapopulation framework. The main message of this review is that HGT should be interpreted as a context-dependent contributor to *L. monocytogenes* adaptation, acting together with clonal background, ecological selection, and mobile genetic elements.

## Simple Summary

*Listeria monocytogenes* is a foodborne bacterium that can cause severe disease in humans and animals, particularly in vulnerable individuals. It persists in diverse environments, including farms, food-processing facilities, soil, and the intestinal tract of animals. Besides the expansion of the clones, its adaptation is influenced by horizontal gene transfer, which can promote the acquisition of antimicrobial resistance and virulence-associated traits. These genetic exchanges may occur across interconnected animal, food, and environmental reservoirs, highlighting the importance of a One Health perspective. This review summarizes the role of horizontal gene transfer in the evolution of *L. monocytogenes* and discusses its relevance for veterinary medicine, food safety, and public health.

## 1. Introduction

*Listeria monocytogenes* is a facultative intracellular, Gram-positive bacterium with a remarkable ecological versatility, inhabiting soil, water, vegetation, wildlife, livestock, and food production environments [1]. It is the etiologic agent of listeriosis, a severe form of invasive disease that in humans manifests as septicemia, meningitis, and maternofetal infections [2,3] and in animals—particularly ruminants—causes encephalitis, abortion, and septicemia [4]. Although the incidence of listeriosis is comparatively low relative to many enteric pathogens, hospitalization and case-fatality rates are disproportionately high, making *L. monocytogenes* a priority for food safety and public health surveillance [5]. The population structure of *L. monocytogenes* comprises distinct phylogenetic lineages and clonal complexes with differential associations to clinical disease, environmental persistence, and food chain dissemination [1,3,6]. While lineage I genotypes are frequently implicated in clinical outbreaks, lineage II genotypes are more common in food and environmental reservoirs [1,6]. The species exhibits a conserved core genome alongside a variable accessory genome enriched in mobile genetic elements, including prophages, plasmids, transposons, and integrative and conjugative elements [1,7,8]. These elements facilitate horizontal gene transfer (HGT), enabling rapid acquisition of adaptive traits. Asymptomatic fecal carriage of *L. monocytogenes* in humans and animals is common and influenced by gut microbiome composition and stability [9,10,11,12]. Under homeostatic conditions, colonization resistance mediated by microbial competition and host mucosal immunity constrains pathogen expansion of infectivity; however, perturbations such as antimicrobial exposure, diet shifts, or stress destabilize microbiota and create niches permissive for transient carriage and shedding [13,14,15]. Although direct experimental evidence of frequent HGT involving *L. monocytogenes* within the gut microbiome remains limited, the high microbial density and shared mobile gene pools suggest that ecological conditions could facilitate occasional genetic exchange events. HGT contributes to the evolution of antimicrobial resistance (AMR) and virulence in *L. monocytogenes*, with mobile genetic elements (MGEs) and accessory genome dynamics playing an important role across ecological compartments [1,7,16,17,18,19]. MGEs mediate gene flow not only within populations but also across species boundaries, facilitating horizontal gene transfer of antimicrobial resistance and other adaptive traits. These elements—including plasmids, transposons, integrons, and bacteriophages—enable the movement of genetic material between distantly related taxa, contributing to the widespread dissemination of resistance determinants and stress-response functions with implications for both food safety and clinical outcomes. MGEs have been shown to mobilize resistance genes across diverse bacterial species and even across phylogenetic boundaries, underscoring their role in shared adaptive repertoires. These evolutionary processes operate within complex metapopulations spanning gut ecosystems, farm environments, processing facilities, and natural habitats, where ecological selection, population bottlenecks, transmission events, and migration drive adaptive diversification. Conceptualizing the evolution of *L. monocytogenes* within eco-evolutionary and metapopulation frameworks provides a useful theoretical perspective for integrating within-host processes with broader transmission networks across farm, environmental, food-processing, and clinical ecosystems. This review integrates current understanding of the mechanisms of HGT in *L. monocytogenes*, examines their roles in antimicrobial resistance and virulence evolution, and explores veterinary and One Health implications of gene flow across hosts and environments. This article is a narrative review based on structured literature searches performed in PubMed, Web of Science, and Scopus. Because this was not designed as a systematic review, no formal PRISMA-based screening, risk-of-bias assessment, or meta-analysis was performed. Priority was given to peer-reviewed articles published between 2000 and 2025, with particular emphasis on genomic, ecological, and evolutionary studies describing population structure, mobile genetic elements, and host–environment transmission dynamics. Previous studies relevant to evolutionary biology and pathogenic mechanisms of *L. monocytogenes* were also included when appropriate. In this review, we adopt a metapopulation framework in which hosts, farms, food-processing facilities, and environmental reservoirs are conceptualized as semi-connected ecological patches linked by migration and gene flow. In this framework, animal hosts, farms, food-processing facilities, natural environments, and humans are considered semi-connected ecological compartments. The framework is used throughout the review to organize how *L. monocytogenes* strains, mobile genetic elements, and selective pressures move between compartments, while local ecological filtering determines whether newly acquired traits are retained, lost, or clinically relevant.

## 2. Population Structure and Genomic Architecture of *Listeria monocytogenes*

**Figure 1:**
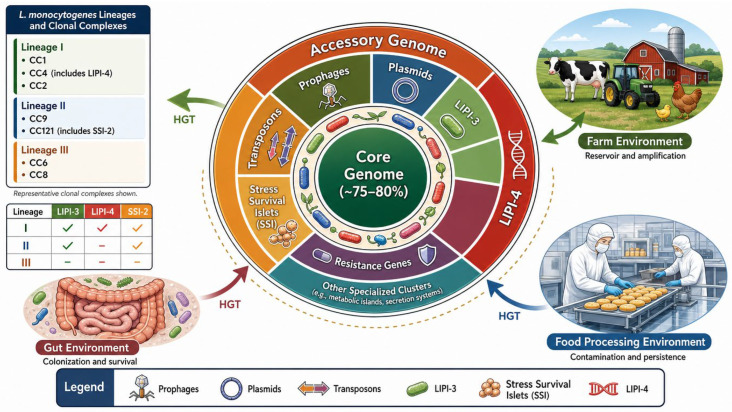
Population structure and genomic architecture of *L. monocytogenes*. The schematic highlights the phylogenetic dichotomy between major lineages and their associated genomic features. The inner circle represents the highly conserved core genome (comprising ~75–80% of coding sequences), which maintains essential metabolic and cellular functions. The outer concentric rings illustrate the accessory genome, a plastic reservoir of prophages, plasmids, transposons, and specialized clusters such as Stress Survival Islets (SSI) and *Listeria* Pathogenicity Islands (LIPI). The distribution of these elements reflects the ecological specialization of specific Clonal Complexes (CCs): Lineage I (e.g., CC1, CC4) is frequently associated with clinical hypervirulence and the presence of LIPI-3/LIPI-4, whereas Lineage II (e.g., CC9, CC121) often carries SSI-2, promoting persistence in food-processing environments. Lineages III and IV are also included as less frequent lineages, predominantly associated with animal hosts and environmental reservoirs, and are represented to provide a complete overview of the four recognized phylogenetic lineages of *L. monocytogenes*. Arrows indicate the continuous gene flow mediated by horizontal gene transfer (HGT) across critical ecological compartments, including the farm environment, food processing facilities, and the host gastrointestinal tract. The figure is intended as a conceptual synthesis of population structure, accessory genome diversity, and ecological interfaces rather than as a quantitative representation of gene flow.

The population structure of *L. monocytogenes* reflects a complex evolutionary history shaped by expansion of the clones, ecological specialization, and genome plasticity driven in part by homologous recombination and horizontal gene transfer (HGT) [1,4,6,19]. Early multilocus enzyme electrophoresis (MLEE) and subsequent multilocus sequence typing (MLST) analyses demonstrated that *L. monocytogenes* is subdivided into four deeply separated phylogenetic lineages (I–IV), which differ in ecological distribution and pathogenic potential [1,20,21]. Lineages I and II are responsible for the majority of human listeriosis cases and are frequently associated with food sources, whereas lineages III and IV are more commonly isolated from animal hosts and environmental reservoirs [1,22,23] (Figure 1).

Whole-genome sequencing (WGS) has refined this framework, demonstrating that *L. monocytogenes* consists of multiple clonal complexes (CCs) nested within lineages, each defined by high core-genome similarity and shared ancestry [8]. Certain CCs exhibit strong ecological or clinical associations. For example, CC1, CC2, CC4, and CC6 (Lineage I) are frequently linked to invasive human disease and are often described as hypervirulent clones [8]. In contrast, CC9 and CC121 (Lineage II) are commonly isolated from food processing environments and display enhanced environmental persistence and reduced virulence potential, illustrating evolutionary trade-offs between environmental adaptation and host invasiveness [24,25] (Table 1).

This section provides the genomic background necessary to understand how clonal structure influences the acquisition, retention, or loss of horizontally acquired elements.

### 2.1. Core Genome Conservation and Recombination Dynamics

However, the impact of mutation and recombination is influenced by the ecological context in which *L. monocytogenes* persists. Population bottlenecks occurring during host colonization may limit genetic diversity, whereas environmental and food-processing reservoirs can support long-term persistence and the accumulation of adaptive genetic changes [18,19,26,27,28]. The widespread distribution of hypervirulent clonal complexes suggests that selective advantages related to host adaptation or transmission may contribute to their expansion [6,8,9]. In addition, dense microbial communities such as gut ecosystems and biofilms may facilitate competition and genetic exchange, further shaping the evolutionary dynamics of *L. monocytogenes*.

### 2.2. Accessory Genome, Pan-Genome Dynamics, and Gene Flow

The accessory genome of *L. monocytogenes* is highly dynamic and includes prophages, plasmids, transposons, genomic islands, stress survival islets (SSIs), and integrative conjugative elements (ICEs) [6,19,29,30]. Pan-genome analyses indicate a moderately open genome, with new genes continuing to be identified as additional isolates are sequenced [6,19]. In a large-scale genomic study of 1696 strains, each newly sequenced genome was estimated to contribute an average of 9.5 unique genes, highlighting the species’ capacity for genetic diversification and environmental adaptation [6].

Prophages represent a major component of the accessory genome and contribute substantially to genome plasticity, phenotypic diversification, and adaptation through lysogenic conversion and the mobilization of accessory traits [31,32,33]. Their distribution varies among lineages, influencing both environmental persistence and pathogenic potential [32,33]. Plasmids are particularly common in food-associated and environmental isolates and frequently carry genes associated with tolerance to heavy metals, disinfectants, and other stresses encountered in food-processing environments [19,25,34,35,36]. Together, these mobile genetic elements facilitate adaptation to diverse ecological niches and reflect the influence of environmental and anthropogenic selective pressures on the evolution of *L. monocytogenes* [37].

### 2.3. Virulence Islands, Selection–Transmission Trade-Offs, and Adaptive Landscapes

Virulence in *L. monocytogenes* is encoded within pathogenicity islands whose distribution varies across lineages. LIPI-1 is conserved in nearly all strains and encodes key determinants required for intracellular infection [38,39]. In contrast, accessory islands such as LIPI-3 and LIPI-4 show a more restricted distribution and are associated with enhanced virulence in specific clonal complexes, particularly CC4 [8,40]. Their mosaic distribution suggests historical horizontal acquisition events followed by clonal expansion, contributing to lineage-specific virulence profiles [7,41].

Genomic plasticity is also reflected in the diversity of internalin genes, which influence host–cell invasion and adaptation to different ecological niches [6,42]. For example, mutations resulting in premature stop codons in internalin gene (*inlA*) are more frequently observed among food-associated isolates and are generally linked to reduced invasiveness, whereas intact alleles predominate in clinical strains [43,44,45]. These patterns highlight the balance between adaptation to environmental persistence and maintenance of virulence traits.

Selection pressures differ across ecological compartments, favoring immune evasion and intracellular replication within hosts, but stress tolerance, disinfectant resistance, and biofilm formation in food-processing environments [37,39,46,47]. Horizontal gene transfer can facilitate adaptation to these distinct conditions by introducing novel virulence- or fitness-associated traits [6,19]. The variable distribution of pathogenicity islands and other virulence determinants across animal, food, environmental, and human isolates highlights the importance of a One Health approach for understanding the emergence, persistence, and transmission of pathogenic *L. monocytogenes* strains.

### 2.4. CRISPR–Cas Systems and Constraints on Horizontal Gene Flow

CRISPR–Cas loci are present in a subset of *L. monocytogenes* strains and contribute to defense against invading mobile genetic elements, thereby constraining the acquisition of foreign DNA and influencing horizontal gene transfer (HGT) dynamics across lineages [48]. Comparative genomic analyses have demonstrated that CRISPR–Cas systems are unevenly distributed among *L. monocytogenes* strains, with substantial variability in locus composition and *cas* gene content, reflecting lineage-specific evolutionary trajectories [49]. This heterogeneous distribution suggests differential selective pressures balancing the benefits of gene acquisition via HGT against the need for phage and plasmid defense [48]. Importantly, CRISPR activity can be modulated or inhibited, for example, by phage-encoded anti-CRISPR proteins identified in *L. monocytogenes*, which effectively relaxes barriers to foreign DNA uptake and promotes horizontal gene flow [50]. Consistent with this, strains exhibiting reduced or inactive CRISPR–Cas systems are expected to experience elevated rates of HGT, facilitating the expansion and diversification of the accessory genome [51]. Collectively, this genomic architecture—characterized by variable CRISPR-mediated immunity and differential accessibility to mobile genetic elements—defines the structural substrate upon which horizontal gene transfer operates.

However, the presence or absence of CRISPR-Cas loci should not be interpreted as a direct quantitative measure of HGT frequency. In *L. monocytogenes*, CRISPR-Cas systems are better regarded as potential modulators of access to foreign DNA, whereas direct experimental evidence linking CRISPR activity to measurable HGT rates remains limited.

By limiting the acquisition of plasmids, prophages, or other mobile genetic elements, CRISPR-Cas systems may also reduce opportunities for the dissemination of antimicrobial resistance determinants, although this effect remains incompletely quantified in *L. monocytogenes*.

## 3. Mechanisms of Horizontal Gene Transfer in *L. monocytogenes*

The available evidence is not equivalent for all HGT mechanisms in *L. monocytogenes*. Conjugation and phage-mediated transduction are supported by stronger experimental and genomic evidence, whereas natural transformation appears to be less prominent, condition-dependent, and less well demonstrated.

**Figure 2:**
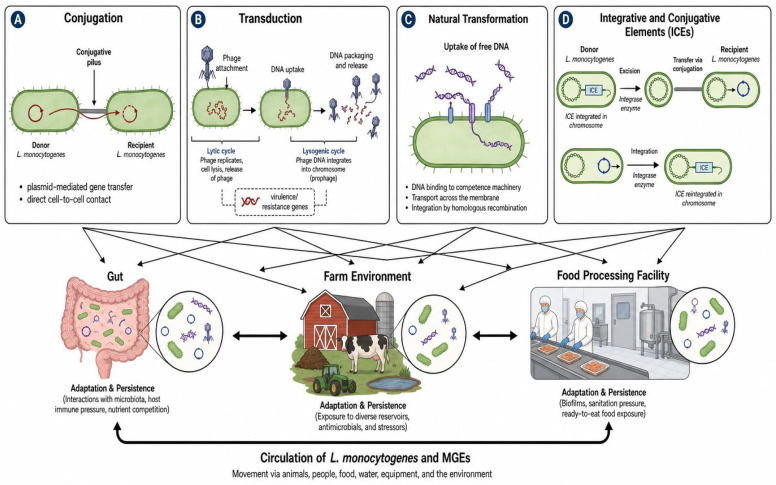
Mechanisms of horizontal gene transfer in *L. monocytogenes*. The illustration details the primary molecular pathways driving genetic exchange and adaptation: (**A**) Conjugation, showing the transfer of single-stranded DNA (ssDNA) from a donor to a recipient cell mediated by plasmid-encoded relaxases and Type IV secretion systems (T4SS); (**B**) Transduction, depicting both lytic and lysogenic cycles where bacteriophages package bacterial DNA into transducing particles, facilitating gene movement between strains; (**C**) Natural Transformation, highlighting the DNA uptake machinery (e.g., Com proteins) involved in the internalization and integration of exogenous genetic material from the environment; and (**D**) Integrative and Conjugative Elements (ICEs), illustrating the excision, circularization, and site-specific integration of these mobile elements into the host chromosome. The lower panel emphasizes that these microscopic processes drive the macroscopic gene flow between the host gut microbiota, farm environments, and food processing biofilms, enabling the rapid dissemination of fitness-enhancing genes across the One Health continuum. Natural transformation is shown as a potential and condition-dependent mechanism. Compared with conjugation and transduction, it is currently less well demonstrated in *L. monocytogenes* and should not be interpreted as equally frequent or equally established.

Horizontal gene transfer (HGT) is a major driver of genome plasticity in *L. monocytogenes*, enabling acquisition of antimicrobial resistance determinants, stress adaptation systems, and virulence-associated loci via mobile genetic elements. Although the species exhibits a largely clonal population structure, its evolution is best described as a predominantly vertical inheritance framework intermittently disrupted by horizontal gene flux, which contributes to genome plasticity and ecological adaptation [52,53,54]. The principal mechanisms contributing to gene exchange include conjugation, transduction, transformation, and mobilization of integrative genetic elements [55,56,57]. HGT drives the evolution of antimicrobial resistance and virulence in *L. monocytogenes* across interconnected ecological compartments [19,58]. Within the gut microbiome, high bacterial density and ecological competition facilitate conjugation, transduction, transformation, and mobilization of integrative elements, promoting the exchange of resistance genes, stress tolerance determinants, and virulence islands [59,60]. These adaptive traits disseminate through fecal shedding into farm environments, food-processing facilities, and human populations, forming a metapopulation network consistent with a One Health framework [61,62]. Eco-evolutionary feedback and selection–transmission trade-offs shape lineage diversification, expansion of the clones, and the emergence of high-risk genotypes [8,63] (Figure 2).

### 3.1. Conjugation

Conjugative transfer represents one of the most important mechanisms for dissemination of antimicrobial resistance and stress adaptation determinants in *L. monocytogenes* [19,55]. Conjugative plasmids encode mobilization (Mob) proteins, including relaxases that initiate DNA transfer by introducing a site-specific nick at the origin of transfer (oriT) [64,65]. The relaxase remains covalently bound to the 5′ end of the transferred strand and guides the single-stranded DNA molecule through a type IV secretion system (T4SS) into recipient cells, where complementary strand synthesis restores plasmid structure. The efficiency of transfer depends on proper assembly of the relaxosome complex, recruitment to the T4SS coupling protein, and stabilization of the transferred DNA within the recipient cytoplasm [59,66,67]. Many plasmids circulating in *L. monocytogenes* encode additional maintenance systems, including partitioning loci and toxin–antitoxin modules, which enhance vertical stability after horizontal acquisition [19,68]. These features are particularly relevant in food processing environments, where exposure to disinfectants and heavy metals imposes selective pressures that favor the persistence of plasmid-bearing strains carrying stress resistance determinants [19,69,70]. Beyond plasmids, integrative and conjugative elements (ICEs), particularly those belonging to the Tn916 family, play a crucial role in the dissemination of antibiotic resistance genes across bacterial populations [71,72,73]. Tn916-like elements integrate into the chromosome via site-specific recombination mediated by a phage-like tyrosine integrase, although other ICE families may encode serine recombinases [73]. Excision is catalyzed by a recombination complex consisting of the integrase and an excisionase, which promotes directional recombination at the element termini [71]. Excision results in the formation of a covalently closed circular intermediate, which constitutes the transferable form of the element during conjugation [73,74]. These elements frequently carry tetracycline resistance genes such as tetM, illustrating a direct mechanistic link between conjugation and antimicrobial resistance spread [75,76]. Within dense microbial ecosystems such as the gastrointestinal tract, physical proximity between bacterial cells and antibiotic-mediated selective pressure may significantly enhance the frequency and persistence of conjugative transfer events [59,77,78].

### 3.2. Transduction

Bacteriophage-mediated transduction is a major driver of horizontal gene transfer and contributes substantially to genomic plasticity in *L. monocytogenes*, facilitating the acquisition of novel genetic traits and promoting evolutionary diversification [79,80]. Temperate bacteriophages infecting this species can adopt either a lytic cycle, characterized by phage replication and host cell lysis, or a lysogenic cycle, in which the phage genome integrates into the bacterial chromosome as a prophage via integrase-mediated site-specific recombination [81,82]. Prophage integration frequently occurs at conserved chromosomal loci, notably within the *com*K gene, which is involved in competence regulation, thereby functionally linking prophage insertion to host regulatory and virulence-associated networks [34,83]. Under stress conditions, including DNA damage and activation of the SOS response, prophages can be induced to excise from the host chromosome via recombinase-mediated processes and re-enter the lytic cycle. During phage assembly, errors in DNA packaging by the terminase complex can lead to generalized transduction, where fragments of bacterial chromosomal DNA are incorporated into phage particles and transferred to new host cells [32]. In contrast, imprecise prophage excision may generate specialized transducing particles carrying adjacent bacterial genes [32,84,85]. Prophages in *L. monocytogenes* are not merely passive genomic elements; they frequently encode accessory functions that influence virulence, stress tolerance, and metabolic capacity [86]. The distribution of lineage-specific prophage repertoires supports ongoing phage–host coevolution and contributes to ecological and phenotypic divergence among clonal complexes [79]. Furthermore, prophage integration can disrupt or modulate host genes, thereby altering transcriptional profiles and influencing bacterial fitness and adaptive potential [34].

### 3.3. Transformation

Natural transformation appears to be less prominent and less experimentally established in *L. monocytogenes* than in naturally competent bacteria such as *Streptococcus pneumoniae*. Although homologues of competence-associated genes have been identified, efficient natural transformation in *L. monocytogenes* seems to be condition-dependent and remains incompletely characterized [87]. Nevertheless, comparative genomic analyses have identified homologues of canonical competence-associated mechanisms in *L. monocytogenes*, including DNA-binding proteins (e.g., ComEA), membrane-associated DNA translocation channels (e.g., ComEC), and recombination systems centered on RecA that mediate homologous integration of exogenous DNA [87,88,89]. Expression of competence genes may be conditionally regulated by environmental stress, nutrient limitation, or growth within structured communities such as biofilms, where regulatory networks (e.g., σ^B-mediated stress responses) play key roles in adaptive physiology [90,91]. Although direct experimental evidence for efficient natural transformation in *L. monocytogenes* remains limited, the conservation of these genetic determinants suggests a latent capacity for DNA uptake under specific ecological conditions [88,89]. This ‘competence-like’ state is not constitutively active but can be triggered by environmental stressors; for instance, the expression of the com machinery is often integrated into broader regulatory networks, such as the σ^B-mediated stress responses, which modulates the physiological transition to competence during nutrient limitation or oxidative stress [90,91]. In structured microbial environments where extracellular DNA accumulates—such as biofilms—prophages can act as active regulatory switches (active lysogeny), where their excision from genes like *com*K restores the functionality of the competence pathway precisely when needed for adaptation [87]. In such contexts, extracellular DNA contributes to both biofilm stability and genetic exchange, promoting diversification within conserved genomic regions.

### 3.4. Integrative Genetic Elements and Genomic Islands

Genomic islands and chromosomally integrated mobile genetic elements substantially expand the evolutionary repertoire of *L. monocytogenes*, contributing to its ecological versatility and pathogenic potential. Comparative genomic analyses reveal that the accessory genome of *L. monocytogenes* is highly dynamic, with frequent acquisition and loss of horizontally transferred elements shaping lineage-specific traits [19,32]. Pathogenicity islands such as LIPI-3 and LIPI-4, as well as stress survival islets (SSI-1 and SSI-2), exhibit discontinuous phylogenetic distributions across lineages, consistent with horizontal gene transfer and subsequent clonal expansion [8,40,92,93]. Structurally, these genomic islands are often flanked by direct repeats and encode site-specific integrases, frequently targeting tRNA loci, which are recognized hotspots for integration [94]. Their integration and excision are typically mediated by tyrosine recombinases or related integrases, while the presence of residual conjugation-associated genes in some islands suggests derivation from ancestral integrative and conjugative elements (ICEs) [73,95]. Functionally, stress survival islets enhance tolerance to acid, bile, osmotic, and oxidative stresses, thereby promoting persistence in gastrointestinal environments and food-processing settings [92,93]. The selective retention of these elements under niche-specific pressures exemplifies how horizontal gene acquisition reshapes bacterial fitness landscapes and drives adaptive evolution [63].

### 3.5. Transposable Elements and Mobilome Dynamics

Insertion sequences (IS elements) and transposons represent additional drivers of genome remodeling. These elements encode transposases that catalyze cut-and-paste or replicative transposition, occasionally forming cointegrate intermediates resolved by site-specific recombinases [96,97,98]. Transposition events can disrupt coding sequences, alter promoter regions, or mobilize adjacent genes, contributing to regulatory variation and phenotypic heterogeneity [59,99]. The cumulative mobilome—plasmids, prophages, ICEs, genomic islands, and insertion sequences—forms a dynamic reservoir of adaptive potential [58]. Table 2 and Table 3 summarizes the main classes of mobile genetic elements contributing to horizontal gene transfer and adaptive diversification in *L. monocytogenes*. Its evolutionary impact depends on ecological context, effective population size, and selective pressures operating across interconnected compartments. In gut ecosystems, high bacterial density and antibiotic exposure may elevate transfer rates. In food processing environments, disinfectant and heavy metal pressures favor retention of tolerance determinants, which can lead to increased survival of certain bacterial strains that may pose risks to food safety and public health. Across metapopulation networks linking farms, wildlife reservoirs, and human hosts, recurrent bottlenecks and migration events determine whether newly acquired genes become fixed, maintained at low frequency, or purged by purifying selection.

The table summarizes the principal classes of mobile genetic elements contributing to genome plasticity in *L. monocytogenes*, their mechanisms of transfer, representative genes or loci, and their ecological or clinical relevance. These elements collectively form the mobilome of the species and play a central role in the dissemination of antimicrobial resistance determinants, stress tolerance traits, and accessory virulence factors across interconnected ecological compartments.

The HGT mechanisms in *L. monocytogenes* function as context-dependent evolutionary accelerators, interacting with clonal inheritance, recombination, and ecological selection to shape antimicrobial resistance evolution and virulence diversification.

## 4. Horizontal Gene Transfer and the Evolution of Antimicrobial Resistance in *L. monocytogenes*: One Health Perspectives

Although *Listeria monocytogenes* has the genetic capacity to acquire antimicrobial resistance determinants through mobile genetic elements, most isolates remain susceptible to first-line treatment regimens used for invasive listeriosis, particularly ampicillin or penicillin combined with gentamicin. Therefore, AMR in this species should not be overstated as a widespread clinical resistance problem. Its importance lies mainly in the ecological and evolutionary potential for resistance gene acquisition, persistence, and dissemination across animal, food-production, environmental, and clinical interfaces. Its resistance profile is supported by a combination of intrinsic and acquired mechanisms, such as active efflux systems, decreased permeability to antimicrobial compounds, enzymatic inactivation, and alterations of antibiotic target sites, all of which reduce drug efficacy. This growing resistance is of particular concern given the role of *L. monocytogenes* in severe foodborne disease outbreaks worldwide. MGEs constitute a fundamental interface between the mobilome and resistome, facilitating the dissemination of AMR genes across interconnected ecological compartments [56,57,100,101,102]. Despite the genetic capacity to acquire antimicrobial resistance determinants through horizontal gene transfer, most *L. monocytogenes* isolates remain susceptible to first-line antimicrobials used in the treatment of invasive listeriosis, particularly β-lactams combined with aminoglycosides. Therefore, the evolutionary significance of resistance in this species lies less in widespread clinical resistance and more in its ecological potential to acquire and disseminate resistance genes across interconnected microbial communities. The emergence of resistant isolates in both clinical and food-production contexts underscores the evolutionary significance of MGEs in shaping the ability of this pathogen to resist. Antimicrobial use in veterinary medicine, food animal production, and human healthcare generates interconnected selective contexts that influence gene acquisition and persistence [14,103,104,105,106]. Animal-associated microbiota may contribute resistance determinants to shared mobile gene pools that *L. monocytogenes* can access through HGT [16].

In this review, intrinsic tolerance refers to native physiological traits that reduce susceptibility without acquisition of foreign resistance genes. Acquired resistance refers to resistance determinants obtained through mobile genetic elements, such as plasmids, transposons, or ICEs. Reduced susceptibility describes measurable decreases in antimicrobial sensitivity that do not always reach clinical resistance breakpoints. Clinically relevant resistance refers to phenotypes that may compromise recommended therapeutic regimens.

### 4.1. Mechanistic Basis of Antimicrobial Resistance Acquisition

Building upon the mechanistic framework of horizontal gene transfer previously discussed, antimicrobial resistance acquisition in *L. monocytogenes* primarily occurs through the mobilization of plasmids and integrative conjugative elements. For instance, *Tn916*-like elements encode the ribosomal protection protein TetM, which confers tetracycline resistance by displacing the antibiotic from the ribosomal A-site without enzymatic modification [17,18,73,76,77,78,79,102,107]. These elements integrate into chromosomes via integrase-mediated, site-specific recombination and are then excised under regulatory control to form circular intermediates competent for conjugative transfer [76,77,78,79]. Macrolide resistance in *L. monocytogenes* appears to be primarily driven by horizontal gene transfer mechanisms involving broad-host-range plasmids. Plasmid *pIP501*, originally described in *Streptococcus agalactiae*, carries macrolide resistance determinants and can be transferred to *L. monocytogenes* via conjugation. Once acquired, this plasmid is capable of stable replication and further dissemination within *Listeria* populations, as well as transfer back to *streptococci*, highlighting the potential for sustained and bidirectional spread of macrolide resistance across genera [102]. Resistance to trimethoprim is primarily associated with the acquisition of alternative dihydrofolate reductase enzymes encoded by MGEs, particularly the *dfr*D gene [7]. Reduced susceptibility to penicillin G has been associated with alterations in penicillin-binding proteins, particularly those involved in peptidoglycan biosynthesis; however, clinically relevant β-lactam resistance remains uncommon in *L. monocytogenes* [108]. The resistance to aminoglycosides, including gentamicin, has been attributed to the presence of MGEs such as transposon Tn3706 on plasmid pIP501, carrying the *aac6′-aph2* gene that encodes enzymes capable of inactivating these antibiotics [17,102]. Although high-level aminoglycoside resistance is infrequent in clinical infections, the genetic capacity for acquisition is present within shared mobile gene pools in the gut microbiota. Resistance to penicillin G has been associated with alterations in penicillin-binding proteins, especially those encoded by the *pen*A gene, which are essential for peptidoglycan biosynthesis [108]. Overall, these findings emphasize the adaptive capacity of *L. monocytogenes* and reinforce the importance of ongoing surveillance and responsible antimicrobial use. Efflux pumps also contribute to intrinsic and acquired resistance phenotypes. Multidrug transporters may enhance tolerance to certain antibiotics [109,110].

### 4.2. Veterinary and Food-Production Selective Pressures

These examples are not direct evidence of HGT. Rather, they illustrate how food-processing and preservation-related stresses may shape ecological selection, stress adaptation, and the retention of mobile elements carrying tolerance determinants.

In livestock and food-processing environments, antimicrobial and preservative pressures shape *L. monocytogenes* ecology and indirectly influence mobile Resistome dynamics. Antimicrobials are used for therapeutic, metaphylactic, and historically growth-promoting purposes in livestock production systems. Although regulatory restrictions have reduced non-therapeutic use in many regions, selective pressure persists in intensive production systems. Tetracyclines, macrolides, and sulfonamides remain widely used in veterinary practice, potentially selecting for mobile resistance determinants transferable to *L. monocytogenes* [106]. The high microbial density and antibiotic exposure within animal gastrointestinal tracts create conditions conducive to conjugative transfer and phage-mediated gene exchange. Consequently, the gut microbiome acts as a reservoir and amplification hub for resistance genes, which can then enter environmental or food-associated *Listeria* populations [111,112]. The food-processing environments represent additional selective niches. Repeated exposure to disinfectants, heavy metals, and sublethal stresses may favor persistence of strains carrying plasmid-encoded tolerance genes [70]. The linkage between cadmium resistance determinants and antimicrobial resistance loci suggests historical co-selection in industrial settings [113]. Specifically, the presence of these determinants on the same mobile genetic elements implies that selective pressures from heavy metals or disinfectants—such as benzalkonium chloride tolerance mediated by Tn6188 [71]—can unintentionally drive the maintenance and spread of antibiotic resistance genes, even in the absence of direct antibiotic exposure. Numerous studies indicate that organic acids and bioprotective cultures can substantially inhibit the growth of *L. monocytogenes* in RTE products, such as meat and fermented salami, underscoring the interaction between environmental and food-derived selective pressures with microbial physiology and gene regulation [114,115,116]. The antimicrobial effect of organic acids in RTE meat products reduces *L. monocytogenes* viability, indicating that natural preservatives may impose selective stress conditions with potential implications for bacterial stress response mechanisms and adaptive persistence [115,117]. Similarly, the use of bioprotective cultures in dry fermented salami reduced the growth potential of *L. monocytogenes,* indicating that microbiome modulation within fermented food matrices can limit pathogen persistence and influence the evolutionary dynamics of stress tolerance [114]. Additionally, shelf-life studies demonstrate that fermented phenolic compounds from parsley root and hawthorn berry phenolics can suppress *L. monocytogenes* growth in RTE Wiener sausages, adding dimension to how natural antimicrobial compounds influence pathogen ecology under cold storage conditions [116]. These findings underscore the importance of ecological context—including food composition, preservation strategy, and storage conditions—in shaping competitive dynamics between *L. monocytogenes* and co-resident microbiota, with downstream effects on HGT opportunities and adaptive gene retention.

### 4.3. Human Clinical Context and Treatment Implications

In humans, invasive listeriosis is typically treated with ampicillin or penicillin in combination with gentamicin. While resistance to β-lactams remains rare, sporadic reports of reduced susceptibility and acquired resistance to alternative agents such as erythromycin or tetracycline highlight the importance of surveillance [76,77,78,79,102,107,112,118]. Importantly, even low-frequency resistance determinants may become clinically relevant if transferred into hypervirulent clonal complexes. The convergence of virulence and resistance genes within the same genomic background would represent a significant public health concern [7,22,119].

### 4.4. Epidemiological Surveillance and Genomic Tracking

Whole-genome sequencing (WGS) has revolutionized surveillance of *L. monocytogenes*, enabling high-resolution tracking of clonal complexes, mobile genetic elements, and resistance determinants across ecological compartments [6,120]. Core-genome MLST (cgMLST) and single-nucleotide polymorphism (SNP)-based phylogenetics facilitate identification of outbreak strains and assessment of gene flow between farm, food, and clinical isolates [121,122]. Resistome analysis through WGS allows detection of plasmid replicons, transposon-associated genes, and integrative elements [6,20,100]. Comparative genomic surveillance has demonstrated that certain resistance determinants cluster within specific clonal complexes associated with food production environments [123]. Integration of phenotypic antimicrobial susceptibility data with genomic characterization from these studies can inform resistome mapping in *L. monocytogenes*, allowing identification of clonal complexes that persist within specific production niches. Such harmonized surveillance—combining international WGS datasets with localized phenotypic and ecological studies—enhances the detection of emergent resistance trends and supports targeted mitigation strategies within veterinary and food-production domains [124].

### 4.5. Evolutionary Implications

The evolution of AMR in *L. monocytogenes* reflects a balance between selective advantage under antimicrobial pressure and potential fitness costs in antibiotic-free environments. Horizontally acquired resistance genes may impose metabolic burdens, but compensatory mutations or co-selection with stress tolerance genes can stabilize their persistence. Across interconnected ecological compartments, gene flow mediated by plasmids, ICEs, and prophages links veterinary, environmental, and clinical domains. The antimicrobial resistance evolution in *L. monocytogenes* cannot be interpreted only as a clinical phenomenon. It is an eco-evolutionary process shaped by agricultural practices, food-processing sanitation regimes, microbial community dynamics, and global trade networks, which collectively influence the spread of antimicrobial resistance across different environments and contribute to the overall virulence of *L. monocytogenes*.

## 5. Horizontal Gene Transfer and Virulence Diversification in *L. monocytogenes*

Virulence diversification in *L. monocytogenes* is shaped by multiple interacting processes, including clonal background, regulatory variation, gene loss, internalin variation, recombination, and, in some cases, horizontally acquired genomic islands. The core virulence mechanisms are chromosomally encoded and vertically inherited; the diversification, modulation, and ecological tuning of virulence traits are strongly influenced by HGT, recombination, and lineage-specific gene flux. These processes operate within structured ecological networks that encompass animal hosts, food production systems, environmental reservoirs, and human populations.

### 5.1. Core Virulence Architecture and Regulatory Networks

The central virulence determinant of *L. monocytogenes* is *Listeria* Pathogenicity Island 1 (LIPI-1), which encodes the transcriptional regulator PrfA together with essential intracellular life cycle factors including listeriolysin O (*hly*), ActA (*act*A), phospholipases (*plc*A, *plc*B), and metalloprotease (*mpl*) [125,126,127]. PrfA functions as the central transcriptional regulator of *L. monocytogenes* virulence, integrating environmental signals such as temperature, carbon source availability, and host-associated stress cues to coordinate the expression of genes required for invasion, phagosomal escape, and actin-based motility [128]. Its activity is tightly controlled through multilayered mechanisms, including RNA thermosensors, carbon catabolite repression, and interaction with stress response pathways such as σ^B, ensuring context-dependent activation of the virulence regulon. Although the *Listeria* pathogenicity island 1 (LIPI-1) is highly conserved across lineages, variation in promoter regions and regulatory elements contributes to differences in virulence gene expression [1,44]. Hypervirulent clones often display enhanced or optimized PrfA activity, whereas food-associated lineages frequently exhibit attenuated virulence gene expression linked to ecological specialization [8]. However, these islands should be interpreted as selected examples of HGT-associated virulence diversification rather than as the sole explanation for virulence differences among clonal complexes.

### 5.2. Horizontally Acquired Pathogenicity Islands

Accessory pathogenicity islands represent key examples of HGT-mediated virulence diversification [20,39]. LIPI-3, encoding listeriolysin S (LLS), is restricted primarily to lineage I strains and is associated with enhanced survival in polymicrobial environments, including the gut microbiome [8,41,129,130,131]. LLS functions as a bacteriocin-like peptide, potentially conferring competitive advantage by inhibiting Gram-positive competitors, thereby influencing colonization dynamics [41,131]. LIPI-4, identified in hypervirulent clonal complexes such as CC4, is associated with neural and placental tropism and has been linked to increased invasiveness in clinical cases [8,132]. Genomic analyses indicate that LIPI-4 was acquired through horizontal transfer and subsequently disseminated via the expansion of the clones [1,8]. Its uneven phylogenetic distribution supports episodic acquisition followed by selective sweeps in host-adapted backgrounds [1,122]. These pathogenicity islands are typically flanked by integrase genes and insertion hotspots, consistent with site-specific recombination mechanisms [129]. Their maintenance suggests positive selection within host environments, while absence in environmentally persistent clones reflects adaptive divergence [1].

### 5.3. Internalin, Surface Proteins, and Selection–Transmission Trade-Offs

The internalin family of surface proteins exemplifies the remarkable virulence plasticity of *L. monocytogenes*. Internalin A (InlA) mediates bacterial entry into intestinal epithelial cells through binding to E-cadherin, thereby facilitating translocation across the intestinal barrier [133,134]. In contrast, Internalin B (InlB) promotes invasion of a broader range of host cell types, including hepatocytes and placental cells, via interaction with the Met receptor tyrosine kinase [135]. Notably, premature stop codons (PMSCs) in the *inl*A gene are frequently identified in food-associated lineage II isolates and are strongly associated with reduced invasiveness in human epithelial cells [136,137]. These truncations are widely interpreted as adaptive modifications associated with ecological specialization. In food-associated environments, the selective pressure to maintain a fully functional *inl*A gene is reduced. Consequently, strains carrying premature stop codons in *inl*A may persist and become prevalent in food-processing settings, while exhibiting reduced adhesion and invasiveness in human epithelial cells [38,133]. From an evolutionary standpoint, this pattern is consistent with a selection–transmission trade-off, whereby strains optimized for invasive disease may incur fitness costs outside the host, while environmentally adapted clones sacrifice invasiveness for improved survival and dissemination in non-host environments. Furthermore, horizontal gene transfer contributes to this adaptive continuum by enabling the acquisition, loss, or modulation of virulence-associated loci, thereby facilitating rapid shifts in pathogenic potential across lineages [8,20,22].

### 5.4. Microbiome-Mediated Selection and Within-Host Evolution

Within the gastrointestinal tract, *L. monocytogenes* encounters intense competition and immune surveillance. The gut microbiome imposes colonization resistance through nutrient competition, bacteriocin production, and immune modulation [15,16]. Horizontally acquired traits such as LIPI-3 may contribute to competitive fitness in polymicrobial environments, although the ecological role of this island in gut microbial competition remains under investigation [41,133]. Antibiotic-induced dysbiosis disrupts these barriers, facilitating pathogen expansion and HGT [14]. In such contexts, mobile genetic elements contribute to the acquisition of resistance and virulence-associated determinants, while high bacterial density and bacteriophage activity further enhance recombination and gene exchange rates [138,139]. Experimental studies demonstrate that *L. monocytogenes* undergoes rapid transcriptional adaptation during host colonization, indicating that short-term evolutionary trajectories may influence infection dynamics and transmission potential [28,140]. Subsequent fecal shedding enables dissemination of microbiome-selected variants into environmental and food-associated reservoirs.

### 5.5. Eco-Evolutionary Implications Across Metapopulations

Virulence evolution in *L. monocytogenes* is best understood within a metapopulation framework linking gut ecosystems, livestock production systems, food-processing environments, and human hosts, where transmission across compartments generates structured but overlapping populations [38,48,141]. Gene flow mediated by HGT connects these ecological niches, while local selective pressures filter adaptive variants and shape lineage-specific genomic repertoires [6,20,23]. In host-associated environments, selection favors traits associated with immune evasion and intracellular replication, including tightly regulated virulence gene expression and metabolic adaptation to the cytosolic niche [28,129,140]. In contrast, food-processing environments impose strong selection for stress tolerance, biofilm formation, and resistance to disinfectants, promoting persistence under sublethal sanitation regimes [142]. Virulence-associated determinants may therefore be retained, attenuated, or lost depending on the relative fitness landscapes encountered across these compartments, reflecting trade-offs between pathogenicity and environmental persistence. Eco-evolutionary feedback arises when genetic changes modify ecological interactions and, in turn, reshape selective pressures acting on bacterial populations. The acquisition of a bacteriocin-encoding island can alter gut microbial community structure and competitive dynamics, thereby influencing opportunities for subsequent HGT events and niche colonization [143]. Conversely, plasmids and transposons conferring disinfectant tolerance enhance persistence in food-processing environments, increasing the probability of transmission along the food chain and ultimately human exposure [71,144]. The virulence diversification in *L. monocytogenes* reflects dynamic interplay between molecular regulation, horizontal acquisition, ecological selection, and transmission structure.

## 6. The Gut Microbiome as an Evolutionary Interface for *L. monocytogenes*

The gut microbiome should be considered a potential ecological interface for *L. monocytogenes* rather than a confirmed major site of frequent HGT. High microbial density, mobile gene pools, bacteriophage activity, and antimicrobial exposure could create conditions permissive for genetic exchange, but direct evidence for frequent HGT involving *L. monocytogenes* in vivo remains limited. In this intricate microbial ecosystem, ecological competition, immune modulation, metabolic cross-feeding, and bacteriophage activity collectively impact colonization dynamics, genetic exchange, and adaptive diversification. In this context, the gut microbiome may act as a selective interface that shapes transient carriage of *L. monocytogenes* and facilitates gene exchange processes relevant to its persistence and evolution.

The gut microbiome may act as a selective interface that shapes transient carriage of *L. monocytogenes* and potentially facilitates gene exchange processes relevant to its persistence and evolution. However, direct experimental evidence demonstrating frequent horizontal gene transfer involving *L. monocytogenes* within the intestinal environment remains limited, and many of the proposed mechanisms are inferred from broader studies of gut microbial ecology, mobile genetic elements, and gene exchange dynamics in complex bacterial communities.

### 6.1. Microbiome Ecology and Colonization Resistance

Although direct experimental evidence for frequent horizontal gene transfer involving *L. monocytogenes* within the gut microbiome remains limited, the intestinal ecosystem represents a potential ecological interface where shared mobile gene pools and selective pressures may facilitate genetic exchange [138]. Under homeostatic conditions, the gut microbiota confers colonization resistance against invading pathogens through nutrient competition, production of short-chain fatty acids, bacteriocins, and modulation of host innate immunity [15,16,145,146]. For *L. monocytogenes*, these mechanisms limit expansion and often result in transient carriage rather than sustained colonization [147,148].

Disruption of microbiome structure—via antibiotic exposure, dietary shifts, stress, or inflammation—reduces ecological competition and increases niche availability [149,150]. Experimental studies demonstrate that antibiotic-induced dysbiosis enhances susceptibility to *Listeria* colonization and systemic dissemination [14]. Such perturbations reduce community diversity and functional redundancy, thereby altering resource landscapes and enabling opportunistic expansion [149].

From an evolutionary perspective, these disturbances transiently increase the effective population size of *L. monocytogenes* within the host, increasing mutation supply and the probability of HGT-mediated gene acquisition [64,151]. Conversely, restoration of microbiome stability imposes renewed selective pressure, potentially purging maladaptive variants [64].

These processes are biologically connected but should not be considered equivalent: colonization resistance limits pathogen expansion, dysbiosis increases niche availability, and HGT represents a distinct genetic process that may occur only under permissive ecological conditions.

### 6.2. Horizontal Gene Exchange Networks in the Gut

Horizontal gene exchange within the gut microbiome occurs in densely populated, multispecies communities in which close physical proximity enhances conjugative transfer of plasmids and integrative conjugative elements [78,138]. These environments sustain a shared pool of mobile genetic elements that function as reservoirs of antibiotic resistance and stress-response determinants, which may be accessed by pathogens, including *L. monocytogenes* [61,152]. In addition to conjugation, bacteriophage-mediated transduction contributes substantially to genomic diversification within gut ecosystems [153]. The high abundance of bacteriophages, coupled with stress-induced prophage activation, increases the likelihood of both generalized and specialized transduction events. Consequently, prophage dynamics in *L. monocytogenes* are likely influenced by broader community-level phage–host interactions and environmental stressors [32,86]. Network-based analyses further demonstrate that horizontal gene transfer (HGT) in the gut microbiome is highly structured rather than random, with certain taxa functioning as hubs for mobile element circulation [61,138]. Within such structured exchange networks, *L. monocytogenes* may function both as a recipient and a donor, integrating into pre-existing gene flow circuits. The likelihood of successful gene acquisition is governed by multiple biological constraints, including compatibility of replication and maintenance systems, restriction–modification barriers, and CRISPR–Cas immune defenses [154,155].

### 6.3. Within-Host Evolution and Adaptive Dynamics

Within-host evolution and adaptive dynamics of *L. monocytogenes* occur over short timescales, driven by selective pressures imposed by host immunity, microbiota competition, and nutrient availability [140]. Experimental infection models demonstrate rapid transcriptional reprogramming and genetic shifts during gastrointestinal transit and systemic dissemination [140,143]. The evolutionary trajectory within a single host may involve clonal interference, where multiple beneficial mutations compete before fixation. Effective population size fluctuates during infection: initial colonization bottlenecks reduce diversity, followed by expansion phases that increase mutational input. Horizontal acquisition of adaptive genes during these windows can shift fitness landscapes, particularly under antibiotic perturbation [20]. Eco-evolutionary feedback arises when adaptive changes in *L. monocytogenes* alter microbial community structure, which in turn modifies selection pressures [16,38]. The acquisition of bacteriocin-associated loci such as LIPI-3 may suppress competing Gram-positive taxa, alter microbial network topology, and potentially influence subsequent gene exchange probabilities [20,143].

### 6.4. Modeling Gut-Associated Evolutionary Dynamics Interface for L. monocytogenes

Mathematical and ecological models of within-host evolution provide a conceptual framework for understanding pathogen persistence and transmission. Metapopulation models conceptualize hosts as interconnected ecological patches linked through migration processes such as fecal shedding and food-chain transmission [156]. In each host patch, mutation, natural selection, genetic drift, and horizontal gene transfer control how evolution works [64]. Selection–transmission trade-offs are particularly relevant. Genotypes optimized for rapid intracellular replication may enhance virulence but reduce transmission opportunities if host morbidity limits shedding duration. Conversely, strains adapted for gut persistence with moderate virulence may achieve greater between-host dissemination, consistent with classical virulence trade-off theory [157]. The gut microbiome mediates these dynamics by influencing colonization resistance, persistence, and pathogen shedding intensity [16,158]. Recent advances in genomic epidemiology and network-based transmission modeling indicate that *L. monocytogenes* strains circulating in livestock-associated gut ecosystems are subject to distinct selective pressures compared with strains in human-associated microbiomes, reflecting differences in antimicrobial exposure, diet, and ecological context [159,160].

### 6.5. One Health Integration: Linking Gut Ecology to Environmental Dissemination

**Figure 3:**
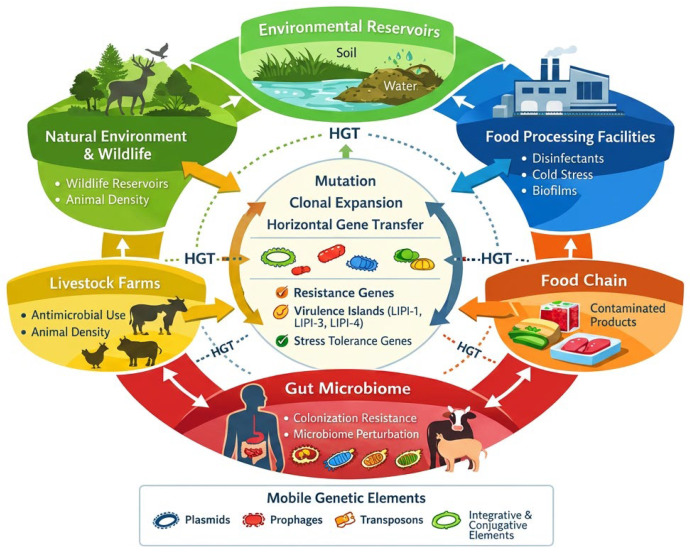
One Health evolutionary network of *Listeria monocytogenes* across interconnected ecological compartments. The diagram illustrates the dynamic flow of genetic information mediated by Horizontal Gene Transfer (HGT), mutation, and clonal expansion. Central to this evolutionary process is the exchange of mobile genetic elements—including plasmids, prophages, transposons, and integrative/conjugative elements (ICEs)—which distribute resistance genes, virulence islands (LIPI-1, LIPI-3, LIPI-4), and stress tolerance determinants. The network highlights the connectivity between six key ecological niches: (1) Natural Environment and Wildlife, serving as primary reservoirs; (2) Environmental Reservoirs (soil/water); (3) Food Processing Facilities, where disinfectants and biofilms drive selection; (4) Food Chain dissemination; (5) Gut Microbiome, as a hub for colonization and genetic exchange; and (6) Livestock Farms, where antimicrobial pressures promote the emergence of mobile resistomes. Bidirectional arrows denote the continuous microbial and genetic connectivity across these compartments, reflecting the One Health perspective on pathogen evolution and dissemination. Specific One Health links include tetracycline resistance genes such as *tetM* carried by Tn916-like elements, disinfectant tolerance genes such as *qacH* associated with Tn6188, cadmium resistance determinants such as *cadA*, and virulence-associated islands such as LIPI-3 and LIPI-4. These elements may be selected by antimicrobial use in animals, disinfectant exposure in food-processing environments, heavy metal contamination, and clinical antimicrobial pressure.

Fecal shedding represents the mechanistic bridge between within-host evolution and environmental dissemination [161,162,163]. Resistant or hypervirulent genotypes selected in the gut can enter farm environments, contaminate food processing facilities, and ultimately reach human consumers [38,48,162]. Environmental persistence then feeds back into livestock exposure, completing a cyclical metapopulation loop [48,164]. Within this integrated framework, the gut microbiome functions as both a selective filter and a genetic exchange hub. Veterinary antimicrobial use, dietary formulation in animal production systems, and other environmental factors shape microbial community composition and may consequently influence the evolutionary dynamics of *L. monocytogenes* [165,166]. Thus, understanding AMR and virulence evolution requires simultaneous consideration of microbiome ecology, molecular HGT mechanisms, and transmission networks operating across interconnected compartments [6,111]. Taken together, these interconnected ecological compartments illustrate how the evolution of *L. monocytogenes* emerges from the interaction between within-host microbial dynamics and broader environmental transmission networks [6,163]. The gastrointestinal microbiome, livestock production systems, food-processing environments, and natural reservoirs form a partially connected ecological network in which bacterial populations and mobile genetic elements circulate across hosts and habitats [6,38,100]. Within this framework, horizontal gene transfer, mutation, and clonal expansion interact with compartment-specific selective pressures—such as antimicrobial exposure, microbial competition, and environmental stress—to shape the persistence and diversification of *L. monocytogenes* populations [95,166,167]. A conceptual overview of these ecological interfaces and evolutionary processes within a One Health framework is presented in Figure 3.

**Figure 4:**
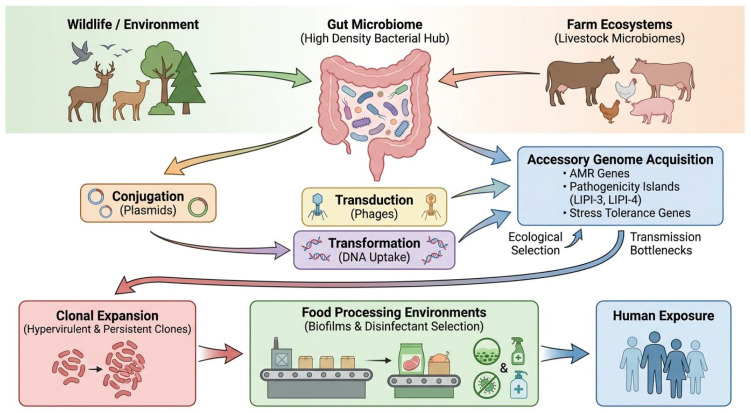
Evolutionary pathways of horizontal gene transfer in *Listeria monocytogenes*. This flowchart delineates the sequential stages and mechanisms of genetic diversification leading to human exposure. The process initiates within diverse reservoirs, such as Wildlife/Environment and Farm Ecosystems, where livestock microbiomes serve as initial sources of genetic variation. The Gut Microbiome acts as a central “high-density bacterial hub”, providing optimal conditions for Horizontal Gene Transfer (HGT) through three primary pathways: Conjugation (plasmid-mediated), Transduction (phage-mediated), and Transformation (extracellular DNA uptake). These mechanisms facilitate Accessory Genome Acquisition, allowing the pathogen to incorporate Antimicrobial Resistance (AMR) genes, Pathogenicity Islands (LIPI-3, LIPI-4), and stress tolerance determinants. The evolutionary trajectory is further shaped by Ecological Selection and Transmission Bottlenecks, leading to the Clonal Expansion of specific hypervirulent and persistent clones. These specialized lineages effectively colonize Food Processing Environments, where they are subject to further selection by biofilms and disinfectants before ultimately reaching the human host through the food chain.

The schematic illustrates how *L. monocytogenes* populations circulate between the gut microbiome of humans and animals, livestock production systems, farm environments, food-processing facilities, and natural environmental reservoirs. Horizontal gene transfer mediated by mobile genetic elements—including plasmids, prophages, transposons, and integrative conjugative elements—facilitates the exchange of antimicrobial resistance determinants, stress tolerance genes, and virulence factors such as pathogenicity islands (LIPI-1, LIPI-3, and LIPI-4). Selective pressures differ across ecological compartments: microbiome-mediated colonization resistance and antibiotic exposure shape within-host dynamics; antimicrobial use and animal density influence farm ecosystems; while disinfectant exposure, cold stress, and biofilm formation affect persistence in food-processing environments. Taken together, these interconnected ecological compartments illustrate how the evolution of *L. monocytogenes* emerges from the interaction between within-host microbial dynamics and broader environmental transmission networks. Figure 4 illustrates the conceptual evolutionary pathways through which horizontal gene transfer connects microbial ecosystems and drives adaptive diversification of *L. monocytogenes* within a One Health framework.

Conceptual model illustrating how horizontal gene transfer mechanisms—including conjugation, bacteriophage-mediated transduction, natural transformation, and integrative element mobilization—facilitate gene exchange across interconnected ecological compartments. The gut microbiome acts as a central evolutionary interface where high bacterial density and ecological competition promote genetic exchange. Mobile genetic elements disseminate antimicrobial resistance determinants, stress tolerance genes, and accessory virulence factors such as pathogenicity islands. Selective pressures across farms, food-processing environments, and host ecosystems shape lineage diversification, clonal expansion, and the emergence of high-risk genotypes within a One Health framework.

## 7. Predictive Surveillance of *L. monocytogenes*

The integration of whole-genome sequencing (WGS), comparative genomics, and high-resolution epidemiological surveillance has transformed the understanding of *L. monocytogenes* population structure and transmission dynamics. Beyond outbreak detection, genomic tools now enable reconstruction of gene flow, mobilome dynamics, AMR dissemination, and virulence evolution across interconnected ecological compartments [6,8,168]. Increasingly, predictive genomics is being explored as a framework linking within-host evolution, food chain contamination, and population-level transmission, although its predictive accuracy remains an area of active development.

WGS has replaced classical subtyping methods, such as Pulsed-field Gel Electrophoresis (PFGE) and Multi-locus Variable Number Tandem Repeat Analysis (MLVA), with nucleotide-level resolution, allowing discrimination between closely related isolates and identification of clonal complexes (CCs) associated with hypervirulence or environmental persistence [6,169]. Core-genome multilocus sequence typing (cgMLST) and SNP-based phylogenetics provide standardized frameworks for international data comparison and outbreak reconstruction [169,170]. Population genomic analyses reveal that lineage I clones (e.g., CC1, CC4) are overrepresented among clinical cases and exhibit genomic signatures associated with hypervirulence, whereas lineage II clones (e.g., CC9, CC121) are more frequently associated with food and environmental persistence [8,38,120]. Increasingly, surveillance datasets incorporate accessory genome characterization, enabling tracking of plasmids, integrative conjugative elements (ICEs), and prophage content. Such analyses identify clusters of isolates sharing resistance determinants or virulence islands, suggesting recent horizontal transfer events [20]. The integration of isolates from animal, food, and environmental sources into WGS surveillance platforms enhances the resolution of transmission pathways linking primary production, processing environments, and human infection. European surveillance systems coordinated through the European Food Safety Authority (EFSA) and the European Centre for Disease Prevention and Control (ECDC) increasingly incorporate genomic data to detect cross-border outbreaks and monitor clonal expansions.

Genomic surveillance enables silico identification of resistance determinants using curated databases such as ResFinder and the Comprehensive Antibiotic Resistance Database (CARD), supporting systematic resistome characterization [171]. Detection of genes such as *tet*M, *erm*B, aminoglycoside-modifying enzymes, and disinfectant tolerance determinants enables early identification of emerging AMR trends [70,113,172]. Beyond single-gene detection, mobilome analysis encompasses plasmid replicons, transposons, integrative elements, and prophages. Comparative genomic studies indicate that specific plasmid backbones are preferably associated with food-related lineages and frequently encode heavy metal resistance, stress response systems, and persistence-associated traits [20]. Mapping these elements across ecological compartments provides insight into gene flow networks and adaptive exchange processes. Phylogenetic incongruence between core and accessory genomes is commonly interpreted as evidence of horizontal gene transfer. Advanced analytical approaches, including Bayesian phylogenetics and recombination-aware models, further refining inference of gene acquisition timing and evolutionary trajectories, although uncertainty remains in resolving directionality in complex transmission networks [173].

### 7.1. Predictive Evolution and Risk Assessment

Predictive evolutionary frameworks aim to integrate genomic, ecological, and epidemiological parameters to estimate the likelihood of emergence and persistence of high-risk genotypes. Models incorporating mutation rates, recombination frequency, effective population size, and selection coefficients provide a quantitative basis for assessing fixation probabilities of newly acquired resistance or virulence determinants. In food-production environments, predictive models evaluate how sanitation practices, biocide usage, and biofilm persistence influence can shape adaptive landscapes by favoring persistence-associated traits and co-selection of linked resistance determinants [48,174]. For example, repeated sublethal disinfectant exposure may increase selection for plasmid-borne tolerance genes, indirectly stabilizing linked resistance determinants. Similarly, antibiotic exposure in livestock may elevate conjugation frequency and enhance persistence of ICE-associated resistance loci. Machine learning approaches applied to large-scale WGS datasets are increasingly used to identify genomic signatures associated with persistence, virulence, or environmental adaptation. However, their predictive performance remains constrained by data heterogeneity and incomplete experimented genotype–phenotype linkage, highlighting the need for integrated phenotypic validation datasets [169].

For example, predictive models could be used to identify clonal complexes carrying combinations of persistence markers, disinfectant tolerance genes, AMR determinants, and virulence-associated islands. Such models may support risk ranking of food-production isolates, early detection of high-risk clones, and prioritization of strains for long-read sequencing or phenotypic antimicrobial susceptibility testing.

### 7.2. Genomic Surveillance Within a One Health Framework

Effective predictive surveillance requires harmonized sampling across human clinical cases, livestock, wildlife reservoirs, food products, and environmental niches. Metapopulation-based models conceptualize these reservoirs as interconnected nodes within a transmission network, enabling estimation of migration rates and identification of source–sink dynamics. Standardization of metadata, including antimicrobial usage patterns, food matrix characteristics, and environmental parameters is critical for contextualizing genomic variation and improving interpretability. Integration of WGS data with quantitative microbial risk assessment models further enables estimation of contamination risk and public health impact under different intervention scenarios [175].

## 8. Conclusions

The evolutionary trajectory of *L. monocytogenes* reflects the interplay between clonal inheritance and episodic horizontal gene transfer within structured ecological networks. Conjugation, transduction, transformation, and mobilization of integrative elements continuously reshape the accessory genome, enabling rapid adaptation to antimicrobial exposure, host immune pressure, and environmental stress.

Virulence diversification results not only from vertical inheritance of core determinants but also from modular acquisition and regulatory tuning of accessory islands, bacteriocin systems, and stress survival loci. Selection–transmission trade-offs drive divergence between hypervirulent clinical clones and environmentally specialized food-associated lineages.

The gut microbiome may represent an ecological interface where *L. monocytogenes* encounters selective pressures, microbial competition, and shared mobile gene pools. However, direct evidence for frequent HGT involving this pathogen within the gut remains limited, and future studies combining long-read sequencing, experimental models, and phenotypic validation are needed.

Advances in whole-genome sequencing and predictive modeling now enable real-time tracking of resistome and mobilome dynamics. However, translating genomic insight into proactive mitigation requires integrated One Health surveillance, ecological modeling, and coordinated antimicrobial stewardship. Moreover, transitioning from the surveillance of particular bacterial lineages to the proactive monitoring of the ‘mobilome’ throughout the food chain may result in more stringent food safety regulations. By prioritizing the surveillance of mobile elements that confer multi-drug resistance or disinfectant tolerance, regulatory bodies can implement targeted interventions in livestock and processing environments before these adaptive traits emerge in clinical settings.

Understanding the evolutionary ecology of *L. monocytogenes* requires an interdisciplinary framework bridging molecular microbiology, evolutionary theory, veterinary science, food safety, and systems epidemiology. Only through such integration can the emergence of high-risk genotypes be anticipated and controlled. Future research that combines comparative genomics, microbiome ecology, and evolutionary modeling will be necessary to predict the rise in high-risk *L. monocytogenes* genotypes and to create effective One Health surveillance strategies.

Future research should focus on combining long-read sequencing, comparative genomics, microbiome studies, and experimental validation to better characterize the mechanisms and ecological drivers of horizontal gene transfer in *L. monocytogenes*. Greater attention should also be directed toward monitoring the mobilome across the food chain, as surveillance of mobile genetic elements may provide earlier warning signals than lineage-based approaches alone. Integrating genomic data with ecological and epidemiological modeling will be essential for predicting the emergence of high-risk genotypes and supporting evidence-based One Health surveillance and intervention strategies.

## Figures and Tables

**Table 1:** Major clonal complexes of *L. monocytogenes*, lineage affiliation, ecological associations, and key genomic features.

Clonal Complex	Lineage	Predominant Ecological Associations	Virulence Profile	Key Genomic Features
CC1	I	Clinical isolates, food, animal reservoirs	Hypervirulent	Intact LIPI-1, frequent LIPI-3
CC4	I	Clinical isolates, maternal-neonatal infections	Hypervirulent	LIPI-4, enhanced neurotropism and placental tropism
CC2	I	Clinical, food, and animal-associated isolates	High virulence	Diverse prophage repertoire
CC6	I	Clinical and food-associated isolates	High virulence	Virulence-associated accessory genes
CC9	II	Food-processing environments, food products, occasional clinical isolates	Reduced virulence	SSI-1, stress adaptation and persistence traits
CC121	II	Food-processing environments and food products	Hypovirulent but highly persistent	SSI-2, frequent inlA PMSCs, disinfectant tolerance
Lineages III–IV CCs	III/IV	Animal and environmental reservoirs	Variable	Diverse accessory genome content

**Table 2:** Actively Mobile Genetic Elements.

Mobile Genetic Element	Mechanism of Transfer	Representative Determinants	Adaptive Function
Conjugative plasmids	Conjugation	*cad*A, *bcr*ABC, *tet*M	AMR, heavy metal and disinfectant tolerance
ICEs (Tn916 family)	Excision + conjugation	*tet*M, *int*, *xis*	AMR dissemination
Prophages	Transduction	integrases, regulatory genes	Genome diversification
Transposons	Transposition	*qac*H, *tet*M	Resistance dissemination
IS elements	Transposition	IS transposases	Genome plasticity

**Table 3:** Horizontally Acquired Genomic Regions (Not Necessarily Mobile).

Genomic Region	Origin	Representative Determinants	Adaptive Function
LIPI-3	Ancient HGT event	lls operon	Enhanced virulence
LIPI-4	Ancient HGT event	cellobiose-family PTS genes	Neural and placental tropism
SSI-1	Horizontally acquired island	stress response genes	Acid and bile tolerance
SSI-2	Horizontally acquired island	stress response genes	Environmental persistence

## Data Availability

No new data were created or analyzed in this study.

## Abbreviations

The following abbreviations are used in this manuscript:

AAC(6′)-APH(2″)	Aminoglycoside Acetyltransferase/Phosphotransferase
AMR	Antimicrobial Resistance
CARD	Comprehensive Antibiotic Resistance Database
CC	Clonal Complex
cgMLST	Core-Genome Multilocus Sequence Typing
CRISPR	Clustered Regularly Interspaced Short Palindromic Repeats
ECDC	European Centre for Disease Prevention and Control
EFSA	European Food Safety Authority
HGT	Horizontal Gene Transfer
ICE	Integrative and Conjugative Element
IS	Insertion Sequence
LIPI	Listeria Pathogenicity Island
LLS	Listeriolysin S
MGE	Mobile Genetic Element
MLEE	Multilocus Enzyme Electrophoresis
MLST	Multilocus Sequence Typing
MLVA	Multilocus Variable Number Tandem Repeat Analysis
Mob	Mobilization Protein
oriT	Origin of Transfer
PFGE	Pulsed-Field Gel Electrophoresis
PMSC	Premature Stop Codon
PrfA	Positive Regulatory Factor A
RecA	Recombinase A
RTE	Ready-to-Eat
SNP	Single Nucleotide Polymorphism
SSI	Stress Survival Islet
ssDNA	Single-Stranded DNA
T4SS	Type IV Secretion System
WGS	Whole-Genome Sequencing
